# P-517. Low Awareness but High Acceptability of HIV Post Exposure Prophylaxis (PEP) among Transpeople Receiving Telemedicine Gender Affirming Care across Rural, Suburban and Urban Areas in the United States

**DOI:** 10.1093/ofid/ofae631.716

**Published:** 2025-01-29

**Authors:** Nancy Aitcheson, Caroline O’Brien, Yun Li, Yixuan Feng, Moira Kyweluk, Florence Momplaisir, Nadia Dowshen

**Affiliations:** University of Pennsylvania, Atlanta, Georgia; Perelman School of Medicine, Philadelphia, Pennsylvania; University of Pennsylvania, Atlanta, Georgia; The Children's Hospital of Philadelphia, Durham, North Carolina; Plume Health, Philadelphia, Pennsylvania; University of Pennsylvania, Atlanta, Georgia; Children’s Hospital of Philadelphia, Philadelphia, Pennsylvania

## Abstract

**Background:**

Transgender people are disproportionately impacted by the HIV epidemic and face unique barriers in accessing HIV prevention services. Post-exposure prophylaxis (PEP), which requires surmounting these barriers within 72 hours of potential HIV exposure, is underused among transgender individuals. Little is known about barriers to using PEP in this population. This survey explored knowledge of and attitudes toward PEP among individuals engaged in gender affirming care (GAHC) via telemedicine.

**Methods:**

We surveyed clients of a telemedicine gender affirming healthcare company in December 2023, using purposeful sampling to ensure representation of all genders, and across geographies. Participants were >18 years old and currently accessing GAHC. Zip codes were classified by urban, suburban, and rural by modified Rural Urban Commuting Score. The online survey asked about knowledge, perceived barriers, and willingness to use PEP. Chi-squared tests were used for statistical comparisons.

**Results:**

Of 387 respondents, 212 identified as transfeminine, 170 as transmasculine and 5 had unknown gender identifications. Participants were spread across geographies of rural (53), suburban (126) and urban (168). Zip codes were missing for 40 participants. 82.7% were white and 51.9% were between the ages of 18-25. 23.8% reported not knowing their HIV status. Awareness of PEP was low among all participants—26.6% had heard of PEP prior to the survey. However, the majority (66.2%) of participants were willing to take PEP if recommended and prescribed by their GAHC providers. Those in rural areas were more likely to be uninsured (37.7%) than those residing in suburban (28.8%) or urban (11.3%) areas (p< 0.001).

**Conclusion:**

Knowledge about PEP was limited among this population, but those seeking GAHC via telemedicine were willing to take PEP and have it prescribed by their GAHC providers. Given the relatively lower rates of insurance coverage, individuals residing in rural areas may experience greater barriers to accessing PEP. Integrating PEP into GAHC may be a strategy to increase timely access to this HIV prevention tool.

**Disclosures:**

**All Authors**: No reported disclosures

